# Image quality in CT thorax: effect of altering reconstruction algorithm and tube load

**DOI:** 10.1093/rpd/ncae005

**Published:** 2024-02-17

**Authors:** Bharti Kataria, Mischa Woisetschläger, Jonas Nilsson Althén, Michael Sandborg, Örjan Smedby

**Affiliations:** Department of Radiology, Linköping University, SE-581 85, Linköping, Sweden; Department of Health, Medicine & Caring Sciences, Linköping University, SE-581 83 Linköping, Sweden; Center for Medical Image Science & Visualisation (CMIV), Linköping University, SE-581 83 Linköping, Sweden; Department of Radiology, Linköping University, SE-581 85, Linköping, Sweden; Department of Health, Medicine & Caring Sciences, Linköping University, SE-581 83 Linköping, Sweden; Center for Medical Image Science & Visualisation (CMIV), Linköping University, SE-581 83 Linköping, Sweden; Department of Health, Medicine & Caring Sciences, Linköping University, SE-581 83 Linköping, Sweden; Department of Medical Physics, Linköping University, SE-581 85 Linköping, Sweden; Department of Health, Medicine & Caring Sciences, Linköping University, SE-581 83 Linköping, Sweden; Center for Medical Image Science & Visualisation (CMIV), Linköping University, SE-581 83 Linköping, Sweden; Department of Medical Physics, Linköping University, SE-581 85 Linköping, Sweden; Department of Biomedical Engineering and Health Systems (MTH), KTH Royal Institute of Technology, SE-141 57 Stockholm, Sweden

## Abstract

Non-linear properties of iterative reconstruction (IR) algorithms can alter image texture. We evaluated the effect of a model-based IR algorithm (advanced modelled iterative reconstruction; ADMIRE) and dose on computed tomography thorax image quality. Dual-source scanner data were acquired at 20, 45 and 65 reference mAs in 20 patients. Images reconstructed with filtered back projection (FBP) and ADMIRE Strengths 3–5 were assessed independently by six radiologists and analysed using an ordinal logistic regression model. For all image criteria studied, the effects of tube load 20 mAs and all ADMIRE strengths were significant (*p* < 0.001) when compared to reference categories 65 mAs and FBP. Increase in tube load from 45 to 65 mAs showed image quality improvement in three of six criteria. Replacing FBP with ADMIRE significantly improves perceived image quality for all criteria studied, potentially permitting a dose reduction of almost 70% without loss in image quality.

## Introduction

The clinical benefits of computed tomography (CT) as a diagnostic tool in medicine support its extensive use in patient care. Thoracic CT is one of the most common CT examinations performed in the radiology department. The thoracic region contains radiation-sensitive organs such as mammary glands, red bone marrow, thyroid glands and lungs^(1)^. Optimisation is thus indicated to meet the clinical objectives of performing CT examinations with the lowest possible radiation dose (as low as reasonably achievable, ALARA) without compromising the diagnostic image quality. The dose can be reduced by selecting automatic tube current (ATCM) and tube potential modulation, by iterative reconstruction (IR) algorithms^(2)^ and by lowering the exposure factors such as tube potential (kilovolt, kV) and tube current time product (tube load, milliampere seconds, mAs). However, reducing the exposure factors (kV and mAs) normally leads to an increase in image noise and thus reduced image quality. Another important factor that influences image quality in CT is low-contrast resolution. Contrast resolution is intrinsically low in CT due to the small differences in attenuation between tissue compositions in the body, and this is especially true for soft tissues for example in the abdomen and brain. To increase soft tissue contrast, contrast agents such as iodine are used to increase contrast resolution^(3)^. However, high inherent contrast between the air-filled lungs and mediastinum in thoracic CT may allow for dose reduction. The noise and artefact suppression properties of IR algorithms are used to optimise protocols. Several research groups have studied the effect of IR algorithms on dose reduction in thoracic CT, where radiation dose reductions as high as 90%^(1, 2, 4)^ are possible. However, the non-linear properties of these algorithms at higher strengths are known to alter image texture and lower diagnostic confidence, thus limiting the potential for imaging at lower doses^(5)^.

Since the thorax is less sensitive to an increase in image noise compared to the abdomen (where low-contrast resolution in the abdomen limits dose reduction), we presumed that thoracic CT can be performed at a much lower dose without affecting the perceived image quality. The aim of the present study was to evaluate how the effect of altering the IR algorithm strength of a model-based IR algorithm (advanced modelled iterative reconstruction, ADMIRE) and dose influences image quality in CT thorax.

## Materials and methods

The Swedish ethical review authority (Diary number: 2015/327/32) approved this prospective study that included 20 patients referred for a clinically indicated CT thorax. The recruited patients were informed of the purpose of the study, and written consent was obtained before the examination was performed.

### Procedure

**Figure 1:**
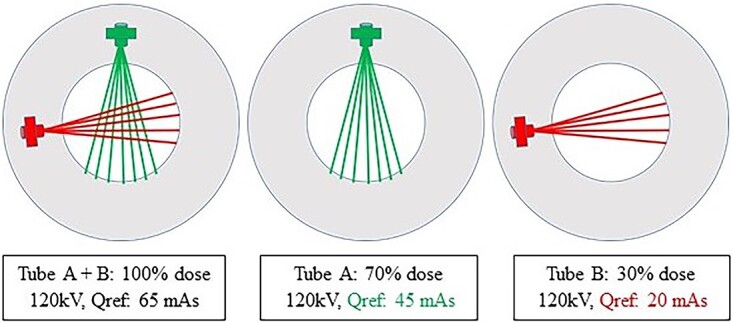
Exposure parameters for the experimental setting on the SOMATOM Force (Siemens Healthineers) used in dual source mode for simultaneous acquisition of three dose levels per patient (adapted and modified with permission from Yoon *et al*.^(9)^).

All examinations were performed on SOMATOM Force (Siemens Healthineers) in the dual-source experimental mode, which enabled simultaneous acquisition of three dose levels per patient obtained by separate reconstruction of images from the two X-ray tube and detector assemblies and a combination of both assemblies without additional exposure to the patient (Figure 1). The quality reference (Qref) values were proportionally equivalent to dose levels of 30% (tube B), 70% (tube A) and 100% dose (tubes A + B). At the time of the study, the clinical standard dose protocol was 65 mAs (100%) dose level with reconstruction algorithm ADMIRE Strength 3. The acquisition parameters for the CT thorax protocol are presented in Table 1. Due to the 35-cm-diameter limitation of the smaller detector, only patients with a body mass index (BMI) of <30 kg/m^2^ (*n* = 20) were included in the study. Patient demographic data such as sex, age, BMI and CT dose predictors, volume computed tomography dose index (CTDI_vol_) and dose length product (DLP) were recorded. All 20 patients underwent a contrast-enhanced thoracic CT acquired in the venous phase at a fixed delay of 40 s.

**Table 1:** Acquisition parameters for SOMATOM Force (Siemens, Erlangen, Germany) for Tubes A and B (smaller detector). Energy composition (DE comp) set at 0.5, i.e. equal kilovolt (kV) weighting for each x-ray tube.

Source	Tube voltage (kV)	Qref (mAs)	Image detector acquisition^a^	Rotation (s)	Pitch	Care dose 4D	Kernel	Dose level	Slice thickness/increment (mm)
Tube A + B	120	65	192 × 0.6	0.5	0.6	Yes	Br36	100%	3/2
Tube A	120	45	192 × 0.6	0.5	0.6	Yes	Br36	70%	3/2
Tube B	120	20	192 × 0.6	0.5	0.6	Yes	Br36	30%	3/2

Qref, quality reference.

^a^57.6 mm detector width.

Non-ionic contrast medium containing iopromide (Ultravist®, Bayer Healthcare) at a concentration of 370 mgI/ml was injected at an individual iodine dose and injection rate based on patient size. The individual dose and injection rate were calculated using OMNIVIS 5.1 (GE Healthcare).

### Visual assessment

**Figure 2:**
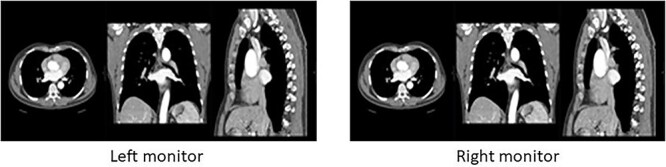
Visual presentation of reading session showing display of multiplanar (MPR) images in axial, coronal and sagittal planes at three different dose levels and four different algorithm strengths, randomly assigned to the right or left monitor.

Multiplanar reconstruction (MPR) images in axial, coronal and sagittal formats at all three dose levels from each patient were reconstructed with filtered back projection (FBP) and ADMIRE strengths of 3, 4 and 5. Visual assessment of image quality, replicating the routine clinical reading (Figure 2), was performed by six radiologists with varying experience (4–25 y) using calibrated monitors (Eizo RX250 monitors, Ishikawa, Japan) on Picture Archive and Communications Systems workstations (PACS, Sectra AB, Linköping Sweden). Anonymised MPR images from the same patient were compared in pairs in a randomised order with random assignment of images at different dose levels and reconstruction algorithms to the right or left monitor.

**Figure 3:**
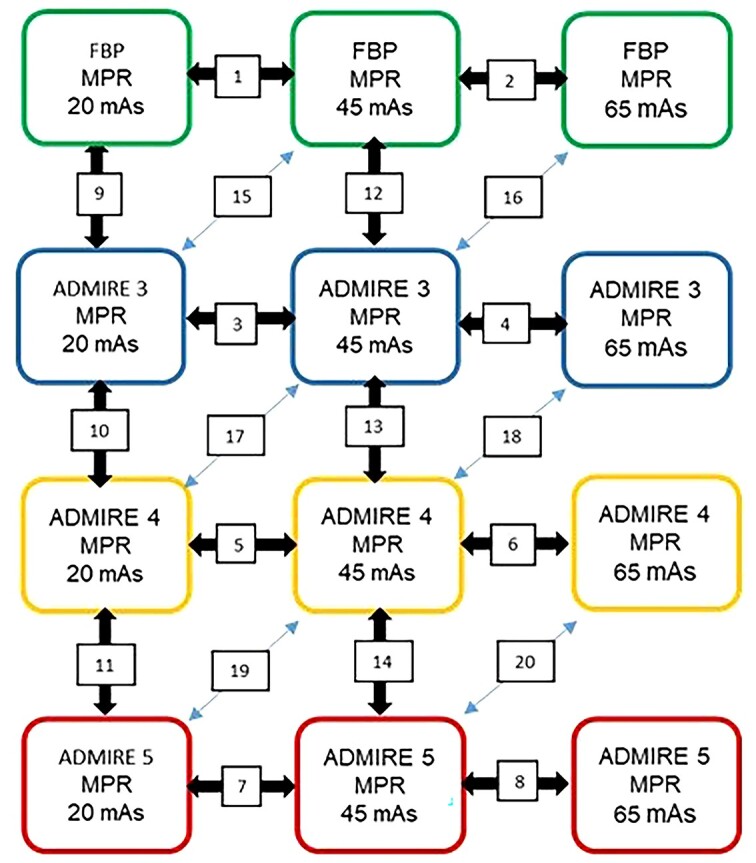
Schematic diagram showing pairwise comparisons of MPR images in the same patient obtained at 20, 45 and 65 mAs reconstructed with FBP and iterative reconstruction (ADMIRE) algorithm at Strengths 3–5.

Figure 3 shows in detail all 20 pairwise comparisons performed in each patient. The criteria used were obtained from European guidelines on quality criteria in computed tomography^(6)^ to suit the purpose of this study, and the images were graded on a 5-point Likert-type scale as shown in Table 2. Prior to the first assessments, all the observers were trained on images (not included in this study) to obtain a similar understanding of the interpretation of the image criteria. When evaluating the criteria, all observers were allowed to change the preferred window settings whenever considered necessary during the reading sessions.

**Table 2:** Image quality criteria (from European guidelines on quality criteria in computed tomography^(6)^) and grading scores.

Image quality criteria:
Criterion 1: visually sharp reproduction of lung parenchyma
Criterion 2: visually sharp reproduction of thoracic aorta and vena cava
Criterion 3: visually sharp reproduction of trachea and main bronchi
Criterion 4: visually sharp reproduction of carina and lymph node area
Criterion 5: visually sharp reproduction of pulmonary vessels
Criterion 6: overall image quality for diagnostic purposes
Grading scores:
−2 Images on left monitor are better than images on right monitor
−1 Images on left monitor are probably better than images on right monitor
0 Images on left and right monitor are equivalent
+1 Images on right monitor are probably better than images on left monitor
+2 Images on right monitor are better than images on left monitor

### Statistical analysis

Statistical analysis was performed within the visual grading regression (VGR) framework^(7, 8)^ using the software R version 4.2.1 (https://www.r-project.org). To study the influence of tube load (mAs) and reconstruction algorithm on image quality scores, a mixed-effects ordinal logistic regression model was defined, including two categorical fixed effects (tube load: 20, 45 or 65 mAs; reconstruction algorithm: FBP, AD3, AD4 or AD5) and two random effects (patient and observer identity) run with the R command *clmm*.

In the statistical analysis, all categories in the respective groups were tested against the reference categories tube load of 65 mAs and FBP reconstruction regardless of which comparisons were actually made by the observers^(8)^. The goodness of fit was reported using McFadden’s pseudo *R*^*2*^^(10)^.

In addition, to estimate the effect of every combination of tube load and reconstruction algorithm, a similar regression model also including interactions between tube load and reconstruction algorithm was applied. In both models, the regression coefficients (effect sizes) describe the variation in image quality due to choice of reconstruction algorithm and tube load. The effect size represents how much the logit of the probability of at least certain score differs from the reference situation FBP and 65 mAs. The significance level was set at *p* = 0.05 with the null hypothesis that there is no difference between the dose levels and reconstruction methods.

To assess how consistent different observers were in their rating of image quality (interobserver reliability), a two-way, mixed, consistency, average-measures intra-class coefficient (ICC) was computed^(11)^. ICC values below 0.40 indicate poor reliability, 0.40–0.59 fair reliability, 0.60–0.74 good reliability and above 0.75 excellent reliability^(11)^.

## Results

The patient population included in this study consisted of 10 males and 10 females, age range 47–92 y (median 76) with a BMI (kg/m^2^) range 16.4–26.8 (median 23). The dose descriptors CTDI_vol_ and DLP ranged from 2.6 to 4.7 (median 3.5) and 89.5 to 165.1 (median 129.7), respectively. Patient demographic data for the study population according to sex are presented in Table 3.

**Table 3:** Patient demographic data presented as range and median for age (years), BMI (kg/m^2^) and dose indices; volume computed tomography dose index (CTDI_vol_, mGy) and dose length product (DLP, mGy.cm) The dose indices below correspond to the 100% dose derived from combined image data of both x-ray tubes.

Sex	Patient descriptor	Range	Median
Female	Age (years)	47–92	77.5
*n* = 10	BMI	16.4–26.8	22.8
	CTDI_vol_	2.6–4.2	3.2
	DLP	89.5–157.2	101.3
Male	Age (years)	49–87	65.0
*n* = 10	BMI	17.6–26.2	23.3
	CTDI_vol_	2.8–4.7	3.7
	DLP	97.6–165.1	134.3

**Figure 4:**
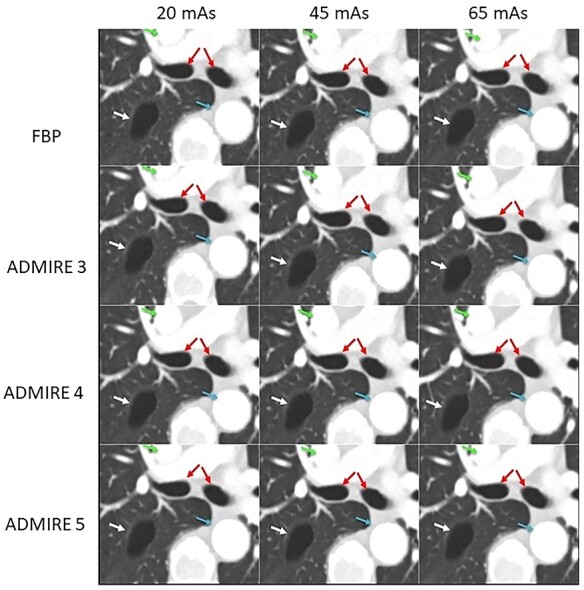
Axial images from a 78-y-old patient at three dose levels reconstructed with FBP and ADMIRE Strength 3–5, showing an emphysematous lesion in the right upper lobe, aorta, superior vena cava and main bronchi. Qref = quality reference.

Representative patient images showing the visual image quality produced by the four reconstruction algorithms at each of the three dose levels are shown in Figure 4. For structures with high intrinsic contrast, such as the contrast-enhanced aorta and superior vena cava, the main bronchi and the emphysematous lesion in the thorax, no major differences in visual subjective image quality can be seen for all dose levels and reconstruction algorithms studied.

**Figure 5:**
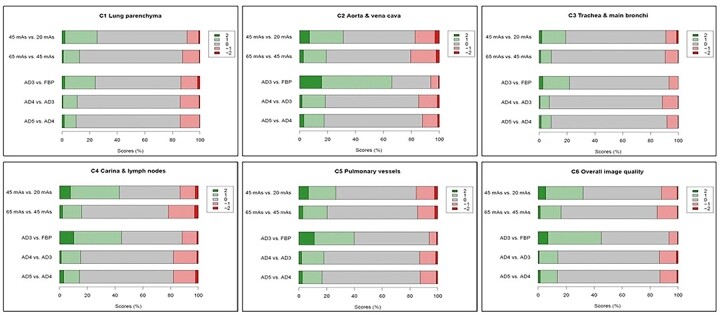
Distribution of image quality scores comparing tube loads and reconstruction algorithms. Positive numbers denote scores favouring the category mentioned first. For image criteria Criterion 1: visually sharp reproduction of lung parenchyma; Criterion 2: visually sharp reproduction of thoracic aorta and vena cava; Criterion 3: visually sharp reproduction of trachea and main bronchi; Criterion 4: visually sharp reproduction of carina and lymph node area; Criterion 5: visually sharp reproduction of pulmonary vessels; Criterion 6: overall image quality for diagnostic purposes.

Scores comparing tube loads and reconstruction algorithms are summarised in Figure 5C1–C6. As seen in the diagrams, a large proportion of image quality comparisons resulted in a score of 0 (grey bars), i.e. both images were rated as equivalent for all criteria. Higher scores for 45 mAs (green bars) than for 20 mAs, as well as higher scores for AD3 (green bars) than for FBP, are visually obvious for all image quality criteria. Differences between 45 and 65 mAs, as well as between the iterative algorithms AD3–AD5, are less evident.

In the regression coefficient analysis (Table 4), the effects of tube load 20 mAs and all reconstruction algorithms were significant (*p* < 0.001) when compared to the reference categories 65 mAs (100% dose) and FBP. However, when comparing the tube loads of 45 and 65 mAs, the regression coefficient is only significant for three of the six criteria. The strongest effects of iterative algorithms were found for Criterion 2 (visually sharp reproduction of thoracic aorta and vena cava) where the corresponding regression coefficient values for AD3–AD5 were 2.48 or higher. These regression coefficient values correspond to the odds of Criterion 2 being more sharply reproduced. For example, when comparing AD3 to FBP, the odds of AD3 obtaining a higher score can be expressed as the exponential, e^2.48^ = 11.9, indicating that AD3 is superior to FBP. For Criteria 2–6, image quality with AD5 was rated significantly higher than that of AD4, which, in turn, was rated significantly higher than that of AD3 for Criteria 1, 2 and 4–6 (see additional significance tests in Table 4).

**Table 4:** VGR based on 2400 comparisons of tube load Qref mAs and reconstruction algorithms FBP, ADMIRE Strength 3 (AD3), ADMIRE Strength 4 (AD4) and ADMIRE Strength 5 (AD5).

Criterion	Regression coefficients (significance tested vs. reference categories 65 mAs and FBP)					Additional significance tests			McFadden pseudo *R*^2^
	20 mAs	45 mAs	AD3	AD4	AD5	20 mAs vs. 45 mAs	AD4 vs. AD3	AD5 vs.AD4	
1. Visually sharp reproduction of lung parenchyma	−0.95^*^^*^^*^	−0.10^n.s.^	0.75^*^^*^^*^	0.99^*^^*^^*^	1.07^*^^*^^*^	^*^ ^*^ ^*^	^*^	n.s.	0.133
2. Visually sharp reproduction of thoracic aorta and vena cava	−1.46^*^^*^^*^	−0.46^*^^*^^*^	2.48^*^^*^^*^	2.96^*^^*^^*^	3.49^*^^*^^*^	^*^ ^*^ ^*^	^*^ ^*^ ^*^	^*^ ^*^ ^*^	0.121
3. Visually sharp reproduction of trachea and main bronchi	−0.93^*^^*^^*^	−0.16^n.s.^	1.12^*^^*^^*^	1.22^*^^*^^*^	1.48^*^^*^^*^	^*^ ^*^ ^*^	n.s.	^*^	0.110
4. Visually sharp reproduction of carina and lymph node area	−1.40^*^^*^^*^	−0.10^n.s.^	1.48^*^^*^^*^	1.76^*^^*^^*^	1.97^*^^*^^*^	^*^ ^*^ ^*^	^*^ ^*^	^*^	0.082
5. Visually sharp reproduction of pulmonary vessels	−1.43^*^^*^^*^	−0.60^*^^*^^*^	1.62^*^^*^^*^	2.13^*^^*^^*^	2.63^*^^*^^*^	^*^ ^*^ ^*^	^*^ ^*^ ^*^	^*^ ^*^ ^*^	0.075
6. Overall image quality for diagnostic purposes	−1.67^*^^*^^*^	−0.46^*^^*^^*^	1.92^*^^*^^*^	2.38^*^^*^^*^	2.81^*^^*^^*^	^*^ ^*^ ^*^	^*^ ^*^ ^*^	^*^ ^*^ ^*^	0.108

^*^^*^^*^p < 0.001; ^*^^*^p < 0.01; ^*^p < 0.05; n.s not significant

In the additional regression analysis, comparison between tube loads 20 and 45 mAs (Table 4) show significant results in favour of the higher tube load for all criteria. Comparisons between IR algorithms AD4 vs. AD3 showed significant results in favour of AD4 for all criteria but one, namely, Criterion 3 (trachea and main bronchi). Similar results are seen for the comparison between AD5 vs. AD4, where all other criteria were significant with the exception of Criterion 1 (lung parenchyma), which was not significant. The McFadden pseudo *R*^2^ values in Table 4 are all <0.2, indicating that the model fit is moderate.

**Figure 6:**
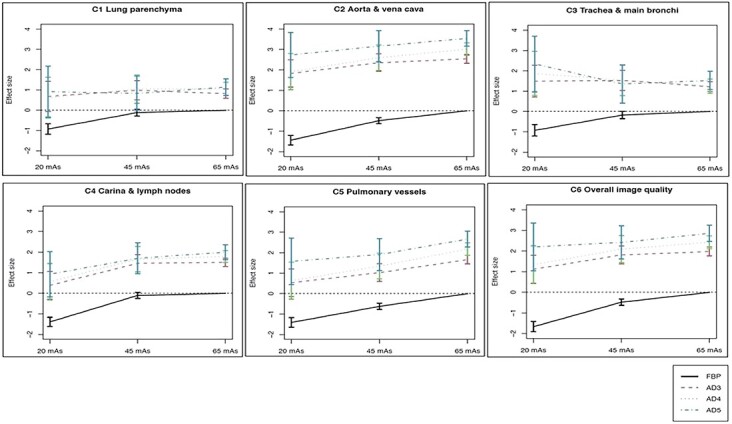
Interaction plots with confidence intervals (error bars) illustrating the effect on image quality scores for combinations of tube load and reconstruction algorithm. The effect sizes shown are expressed on the same scale as the regression coefficients in Table 4, i.e. they represent how much the logit of the probability of the score exceeding a certain grade differs from the reference situation FBP and 65 mAs (higher values correspond to higher rated image quality). The dashed horizontal line represents an odds of e^0^ = 1, i.e. an unchanged probability of at least a given score. Criterion 1: visually sharp reproduction of lung parenchyma; Criterion 2: visually sharp reproduction of thoracic aorta and vena cava; Criterion 3: visually sharp reproduction of trachea and main bronchi; Criterion 4: visually sharp reproduction of carina and lymph node area; Criterion 5: visually sharp reproduction of pulmonary vessels; Criterion 6: Overall image quality for diagnostic purposes.

Effect size estimations of specific combinations of tube load and reconstruction algorithm (on the same scale as the regression coefficients) are found in Figure 6C1–C6. By definition, the reference categories, i.e. FBP and 65 mAs, have a regression coefficient or effect size of zero. A negative effect size thus indicates inferior image quality and a positive effect size, superior image quality. 

 For a majority of criteria, the image quality with all iterative reconstruction algorithms (AD3–AD5) at all studied tube loads (20, 45 and 65 mAs) was rated higher or equal to that of FBP at 65 mAs (Figure 6). In particular, large differences between reconstruction algorithms were seen for Criteria 2 and 4–6. For Criterion 3, the trend was less clear, but image quality rather close to that of FBP at 45 or 65 mAs was found for all the iterative algorithms, regardless of the tube load. For Criterion 1, lung parenchyma, the image quality for AD3–AD5 at all three tube loads was similar.

For the interobserver reliability, the ICC values (Table 5) ranged from poor (ICC = 0.35–0.36) to fair (0.55) to good (0.63–0.74), with the highest value for Criterion 2 (thoracic aorta and vena cava).

**Table 5:** Inter-observer reliability expressed as intraclass correlation coefficient, ICC.

Criterion	Intra-class correlation coefficient (95% confidence limits)
1. Visually sharp reproduction of lung parenchyma	0.356
	(0.253; 0.449)
2. Visually sharp reproduction of thoracic aorta and vena cava	0.742
	(0.700; 0.779)
3. Visually sharp reproduction of trachea and main bronchi	0.346
	(0.700; 0.779)
4. Visually sharp reproduction of carina and lymph node area	0.631
	(0.572; 0.685)
5. Visually sharp reproduction of pulmonary vessels	0.551
	(0.479; 0.616)
6. Overall image quality for diagnostic purposes	0.657
	(0.601; 0.707)

## Discussion

This prospective study evaluated the influence of radiation dose and iterative reconstruction algorithms on image quality in thoracic CT. It is apparent that the benefits of CT in medical imaging outweigh the risks. However, for CT technique to reach its true potential, dose optimisation in accordance with the ‘as low as reasonably achievable’ ALARA principle is paramount since thoracic CT involves irradiation of organs such as breasts, red bone marrow and lungs that are sensitive to radiation^(12)^. In the present study, for all dose-level comparisons, a large proportion of the observer scores were predominantly ‘equivalent’ image quality for all pairwise comparisons performed. These results indicate that radiation dose does not have a strong influence on image quality (delineation and reproduction of image criteria) in thoracic CT. The achievable dose reduction might be correlated to the high intrinsic tissue contrast between the air-filled lungs, lesions and vessels^(2, 13)^.

In the present study, highly significant regression coefficients indicated improvement in image quality (delineation and reproduction of image criteria) when comparing 65 to 20 mAs. However, when comparing 65 to 45 mAs, a significant improvement in image quality is seen for only three of six image criteria assessed.

Hence, even without changing the reconstruction algorithm, a reduction of the tube load from 65 to 45 mAs (by ~30%) may leave many aspects of image quality unchanged. On the other hand, Figure 6 suggests that replacing FBP with any of the iterative reconstruction algorithms may enable us to reduce the tube load from 65 to 20 mAs without losing image quality, i.e. a reduction of the effective dose by almost 70%. This is in accordance with the findings of Jensen *et al*.^(5)^ who investigated the performance of AD3. They found that regression coefficients for the majority of anatomical criteria, lesion conspicuity and noise impression were significant only for middle doses (CTDI_vol_ 4 mGy) when compared to low doses (CTDI_vol_ 1 mGy) and high doses (CTDI_vol_ 6 mGy). However, the dose levels evaluated in the present study (standard dose (100%) CTDI_vol_ between 2.6 and 4.7 mGy) are lower than those used by Jensen *et al*.^(5)^.

IR algorithms have the ability to preserve spatial resolution in the CT images, which is critical for applications of this technique in thoracic CT^(13)^. Additionally, the noise reduction properties of IR allow for substantial dose reductions with preserved image quality^(13)^. Dose reduction is particularly beneficial for dose optimisation in young patients and those who require repetitive imaging. Such dose reductions can be achieved in clinical practice with the application of low-dose (LDCT) and ultralow-dose (ULD) scan protocols achieved through specific adjustment of technical scan parameters (to lower the dose), use of IR algorithms (to compensate for the increase in noise and artefacts) and tin filtration^(12)^. Previous literature has shown that the image quality in ULD thoracic CT is unaffected when using IR^(14)^.

This has facilitated the introduction and use of LDCT protocols for a number of clinical indications in thoracic CT such as lymphoma, COVID-19 pneumonia, pulmonary emphysema, bronchiolitis and oncology surveillance staging^(12, 15, 16)^. Suliman *et al*.^(12)^ conducted a survey on the use of LDCT and ULD in thoracic CT compared to standard dose (STD) protocols in the diagnosis, treatment and follow-up of COVID-19 pneumonia patients. Their results show that the cumulative dose burden of performing a series of CT examinations in the treatment and follow-up of COVID-19 on the same patient can be reduced by a factor of 2–4 when using LDCT and by a factor of 8–13 with the use of ULD. Similarly, often young patients, with bronchiolitis (due to high recurrence rate) and pulmonary emphysema (due to therapeutic evaluation) are subjected to additional acquisitions and repetitive imaging. The findings of the present study are in line with Bankier *et al*.’s study^(15)^ where low-dose protocols (at 20 mAs) have shown to have no substantial effect on visual quantification of air trapping in the former and provide valid measurements of lung parenchymal destruction and growth at follow-up after treatment in the latter. Yoon *et al*. studied the effect of dose and image quality in abdominal CT patients with lymphoma. Considering the long period of surveillance, their results suggest that a LDCT and/or a ULD CT can safely replace a STD CT for the follow-up assessment of lymph-node enlargement in lymphoma patients^(9)^. Pauchard *et al*.^(16)^ compared model-based (MBIR) and hybrid IR algorithms (from the same vendor) in LDCT to determine which algorithm provides the best image quality in young oncology patients. Their findings indicate that the MBIR provides superior image quality compared to the hybrid IR, despite the pixelated appearance of images produced by the former. They recommend that the MBIR be used for staging in young oncology patients to reduce the radiation burden for this patient group. In their study, the MBIR also maintained image quality in LDCT for patients with a higher BMI.

Paolini *et al*.^(17)^ investigated the effect of IR algorithms together with the added benefit of contrast enhancement in LDCT. Their study compared unenhanced LDCT (UN-LDCT) to contrast-enhanced LDCT (CE-LDCT) with the added value of combining axial and coronal reconstructions in lymph node delineation. The results of their study imply that CE-LDCT is superior in delineation of mediastinal lymph nodes compared to UN-LDCT. The results of the present study indicate how IR algorithms may provide the necessary dose reduction for LDCT and could be beneficial in CE-LDCT chest imaging for improved delineation of mediastinal structures compared to FBP^(17)^. CE is known to improve image quality, as illustrated by the highest significant effect on overall image quality (Criterion 6) and the delineation of CE-large vessels (Criteria 2 and 5) in the present study. This improvement in image quality may be crucial in the detection of intrathoracic lymphadenopathy and its distinction from other tissues in the thorax^(17)^.

The presence of small focal lesions and thin vessels in the thorax together with the high intrinsic contrast resolution of the lung parenchyma imply that spatial resolution is more important than low-contrast resolution in thoracic CT. Thus, image texture changes, which are typical for higher strengths of the IR, do not affect image quality in thoracic CT to such a great extent^(5)^. This is evident from the results of the present study, which showed improved image quality in relation to FBP with increasing algorithm strength for all criteria (Table 4). In the additional analysis, when comparing AD4 to AD3, significant image quality improvement was seen for almost all assessed criteria except one, i.e. Criterion 3 (trachea and main bronchi). The diverse results for Criterion 3 (trachea and bronchi) could be explained by motion artefacts related to respiratory or cardiac activity seen in some patient images. Comparing AD5 to AD4, image quality was significantly improved for all criteria except Criterion 1 (lung parenchyma). The poor performance of AD5 for Criterion 1 (lung parenchyma) can possibly be explained by the typical ‘blotchy’ image appearance in higher strengths of ADMIRE that may hide small structures^(5)^. Despite the high intrinsic contrast of Criterion 1 (lung parenchyma) and Criterion 3 (trachea and bronchi), the effect size of the various IR algorithm strengths is small at all dose levels. In other words, the choice of algorithm strength does not seem to have a large influence on the rated image quality, compared to the choice of ADMIRE rather than FBP or the dose level.

IR algorithms may affect other image quality parameters such as low-contrast resolution, spatial resolution and noise structure. Image texture changes can be seen with increasing algorithm strength due to the left shift of the peak frequency towards the lower spatial frequencies in the noise power spectrum (NPS). This shift affects the low-contrast resolution and diagnostic efficacy and limits dose reduction possibilities in abdominal CT^(18)^. In thoracic CT, Jensen *et al*.^(5)^ showed that the NPS peak frequency (although with a slight drop in magnitude at 1 mGy) remained at the same level for ADMIRE 3 when compared to FBP. However, they found a small increase in low-frequency noise at 1 mGy^(5)^. This may explain the lower performance of this particular IR algorithm at low-dose levels. Additionally, a drop in the peak frequency of the NPS results in a coarser noise texture that may affect the visual image impression and possibly diagnostic efficacy^(13)^.

A clear advantage of our study design was the simultaneous acquisition of all dose levels on the same individual, thus avoiding ethical issues of unnecessary radiation exposure to the patients included in this study. The advantage of obtaining all dose levels in the same breath hold also provided the same contrast enhancement phase for a more accurate comparison. If the different dose series are obtained separately, coregistration of the anatomy is not always possible and subtle differences in contrast enhancement need to be taken into account when accessing the image quality^(19)^.

### Limitations

There are several study limitations. The first is that the results apply only to patients with BMI < 30 due to detector length restrictions of the shorter CT detector. Dose reductions in patients with a higher BMI can be challenging. The results are also specific for image quality produced by the ADMIRE algorithm and cannot be applied to other vendor modalities. The subjective image quality evaluation does not necessarily reflect on the diagnostic performance. Therefore, future research involving lesion detection tasks, e.g. receiver operating characteristic (ROC) curve analysis, is indicated to evaluate the clinical implications of IR algorithms on image quality, lesion detection and subsequent dose optimisation.

## Conclusion

ADMIRE showed improved image quality compared to FBP. Replacing FBP with any of the ADMIRE algorithms might permit a reduction of the effective dose by almost 70% with unchanged or improved image quality. Improvements in perceived image quality between ADMIRE Strengths 3, 4 and 5 are significant for most criteria and in particular for thoracic aorta and vena cava, pulmonary vessels and overall image quality, but less so for lung parenchyma and trachea and main bronchi. ROC studies are indicated to explore whether further dose reduction with ADMIRE is possible whilst maintaining clinically acceptable image quality.
